# The University of Buffalo

**Published:** 1911-05

**Authors:** 


**A Monthly Review of Medicine and Surgery**

*EDITOR*
WILLIAM WARREN POTTER, M. D.

All communications, whether of a literary or business nature, books for review and exchanges, should be addressed to the editor, 238 DELAWARE AVENUE, BUFFALO, N.Y.

VOL. LXVI. MAY, 1911. No. 10

### The University of Buffalo

THE effort to establish a liberal arts department of the University of Buffalo by the appropriation of $75,000 a year by the city of Buffalo for a period of twenty-five years has been defeated in the board of aldermen who, on April 17, voted to "receive and file" a resolution providing "that the Common Council of the city of Buffalo do now go on record as being willing to enter into a suitable contract with the University of Buffalo for the purposes specified in the enabling act." "Receiving and filing" is a polite way of announcing the death of a resolution in the board of aldermen.

This is the last move and, for a time at least, eliminates hope of city aid for the greater university project. Were this a purely economic proposition and the city in such financial condition as to make any appropriation for the higher education of its high school graduates impossible, there would be some measure of excuse for the attitude of those fifteen single-purposed gentlemen who voted to shelve the resolution. But the city is not poor; it is merely afflicted with a group of power-ridden aldermen who wide-eyed to their own advantage are deeply bitten of fanaticism and blind obedience to those influences which were drawn into the fight against the extension of the University of Buffalo under any circumstances whatever.

The history of this struggle is too recent of enactment to need more than a brief resumé. The proposition was tendered to the city that if such appropriation were made there would be given in return a certain number of free scholarships to the city and county. There appeared to be little objection to the plan, there being apparently no bar to its successful issue, save that of the necessary conferences to provide such protection to both the university and the city as would make the exchange of education for money equable. Then arose a factor born of personal enmity and spite against a member of the Council of the University and a warfare of venomous activity and disgraceful falsity was instituted against the proposition, the university and its council, the latter were accused of being "cheap grafters" and selfish "money hunters." There came to the surface in this attack, in the course of time, a figure of one whose personal vanity resented a disciplinary action at the hands of the faculty of the university. Just how and by what manner of necromancy it became possible to inject into the question a religious issue is not known. The fact that there did appear religious opposition, veiled in the beginning but none the less fixed, became apparent with the issuing of calls for mass meetings to oppose extension of the university in any form with city aid and this religious opposition was fostered and urged by various means until it became forceful and its effect was readable in the actions of those of the members of the board of aldermen who were directly under its influence. The issue was then brought fairly and squarely into the open. It became a question of merely as to whether or not the aldermen would consider in any form a university proposition looking to the city for aid of financial character. The shifting attitude of the aldermen of easy control and the real opposition were apparent from the beginning. The opponents at first declared in favor of the university provided the city control the institution. This was in a measure granted and then a new demand was made; the whole matter should be referred to the people. This, too, was agreed upon and when put to the test the single purpose of those bed-ridden aldermen, under the leadership of the only member of the present board who may properly be termed an alderman of the old school, was displayed and they declared themselves as opposed to a university extension under any circumstances whatever.

The final test came when Alderman Bruso introduced the resolution mentioned above. There were fifteen votes against the resolution and eight for it. Those who voted against it were Aldermen Bauer, Bradley, Britz, Burley, Costello, Harris, Kavany, Kelberer, Leninger, Metzler, Nowak, Schilling, Shifferns, Edward Stengel and Sullivan. The votes in favor of the resolution were by Aldermen Bruso, Coppins, Eberle, Haffa, Humphrey, Staples, Sam Stengel and Weimar.

And with one exception the vote was the same on the question whether the matter should be referred to the people at the polls.

One of the aldermen, Costello, read a portion of the introduction to Dr. Roswell Park's History of Medicine as a reason for his vote against the University and stated:

"I believe that book is sectarian, bigoted, libelous, scandalous and injurious to the people I represent and I cannot vote for any contract with the University of Buffalo."

Incidentally, it might be interesting to know who presented this alderman with this delicate bit of false argument.

This aldermanic action is in nowise a definite defeat for the project of providing the city of Buffalo with an arts department of the University of Buffalo. It is merely the expression of fifteen men of shifty impulse who have been hypocritic in their objections and who when forced into the open have declared themselves against the establishment of a liberal arts department under any conditions whatever except those of a city built, a city conducted and city controlled college; and were this conceded they would still be found voting against a greater university.

When the clouds of misrepresentation which have enveloped the proposal from the very first are dispelled, it will be found that the basis of voiced objection to the proposed university extension rests wholly on opposition to the teaching of what is generally and universally termed a materialistic or an atheistic philosophy. That is all.

All the talk of "graft" and "money grabbing" for the purpose of "building up decaying and tottering departments" already in existence is not only false but malicious and deliberate in its falsity.

That religion should have been dragged into the controversy and made the catspaw of individual spite and effort toward petty personal revenge is disgraceful. That religion should have permitted itself to be placed in this pitiable, humiliating and undignified position is regretable. Religion is too serious, too beautiful a belief, to be besmirched with the reckless and selfish mouthings of any individual or group of individuals self-centered in their pursuits of personal vengeance and that it should have permitted itself to be placed in this pitiable, humiliating and undignified position is regretable.

